# Muscle Stem Cell-Derived Extracellular Vesicles Reverse Hydrogen Peroxide-Induced Mitochondrial Dysfunction in Mouse Myotubes

**DOI:** 10.3390/cells9122544

**Published:** 2020-11-26

**Authors:** Kyle T. Shuler, Brittany E. Wilson, Eric R. Muñoz, Andrew D. Mitchell, Joshua T. Selsby, Matthew B. Hudson

**Affiliations:** 1 Department of Kinesiology and Applied Physiology, University of Delaware, 540 S College Ave, Newark, DE 19713, USA; kshuler@udel.edu (K.T.S.); bewilson@udel.edu (B.E.W.); emunoz@udel.edu (E.R.M.); andmit@udel.edu (A.D.M.); 2 Department of Animal Science, Iowa State University, 2356G Kildee Hall, Ames, IA 50011, USA; jselsby@iastate.edu

**Keywords:** muscle stem cells, extracellular vesicles, muscular dystrophy, cachexia, oxidative stress, skeletal muscle, mitochondria

## Abstract

Muscle stem cells (MuSCs) hold great potential as a regenerative therapeutic but have met numerous challenges in treating systemic muscle diseases. Muscle stem cell-derived extracellular vesicles (MuSC-EVs) may overcome these limitations. We assessed the number and size distribution of extracellular vesicles (EVs) released by MuSCs ex vivo, determined the extent to which MuSC-EVs deliver molecular cargo to myotubes in vitro, and quantified MuSC-EV-mediated restoration of mitochondrial function following oxidative injury. MuSCs released an abundance of EVs in culture. MuSC-EVs delivered protein cargo into myotubes within 2 h of incubation. Fluorescent labeling of intracellular mitochondria showed co-localization of delivered protein and mitochondria. Oxidatively injured myotubes demonstrated a significant decline in maximal oxygen consumption rate and spare respiratory capacity relative to untreated myotubes. Remarkably, subsequent treatment with MuSC-EVs significantly improved maximal oxygen consumption rate and spare respiratory capacity relative to the myotubes that were damaged but received no subsequent treatment. Surprisingly, MuSC-EVs did not affect mitochondrial function in undamaged myotubes, suggesting the cargo delivered is able to repair but does not expand the existing mitochondrial network. These data demonstrate that MuSC-EVs rapidly deliver proteins into myotubes, a portion of which co-localizes with mitochondria, and reverses mitochondria dysfunction in oxidatively-damaged myotubes.

## 1. Introduction

Muscle stem cell-derived extracellular vesicles (MuSC-EVs) play a central role in skeletal muscle repair and remodeling [1]. Upon injury to muscle, muscle stem cells (MuSCs) are activated, proliferate, and differentiate into myoblasts, which fuse into the preexisting myotubes to facilitate regeneration. Due to their role in regeneration and repair, MuSCs may provide therapeutic benefit to a range of muscle disorders and pathologies [2,3]. Although current strategies in MuSC-based therapies have focused primarily on the isolation, culture, and transplantation of MuSCs in the treatment of muscular dystrophies, these cells may also hold therapeutic potential in numerous other muscle pathologies including cachexia, sarcopenia, mechanical ventilation, disuse muscular atrophy, and muscle trauma [4,5,6,7,8,9,10,11,12,13]. Although initially promising, there are limitations to a transplantation-based strategy [14,15]. For example, since skeletal muscle is the most abundant tissue in humans, a very high number of MuSCs are likely required to treat systemic muscle conditions, though the number of MuSCs required to achieve a lasting therapeutic benefit is equivocal [2]. Further, generating the large number of MuSCs needed for therapeutics requires expanding MuSCs ex vivo; however, the capacity to expand isolated MuSCs is limited before myogenic potential rapidly declines [16].

Extracellular vesicles (EVs) are small, lipid-coated vesicles that contain molecular cargo and are generally classified into overlapping size ranges as exosomes (30–150 nm), microvesicles (100–800 nm), and large EVs (apoptotic bodies, 200 nm–5 µm), and contain a variety of molecular cargo including protein, RNA (including microRNA), and DNA [17,18,19]. EVs participate in paracrine and endocrine-like signaling via the selective packaging of specific molecular cargo that are delivered into nearby and distant recipient cells [20]. Most, if not all, cell types including muscle cells, release EVs [18,21]. Recent evidence indicates EVs play critical roles in allowing MuSCs to respond to stimuli as well as interact with other cell types within the muscle niche [22,23,24]. Murach et al. (2020) demonstrated that satellite cells (Pax7+ MuSCs) release EVs during load-induced hypertrophy to communicate with muscle cells and facilitate extracellular matrix remodeling [25]. Fang et al. (2020) showed that MuSCs instruct macrophages to acquire an anti-inflammatory phenotype via the release of insulin-like growth factor-2 and MuSC-conditioned media relieves inflammatory bowel disease in mice [26]. Additionally, EVs released by cardiosphere-derived cells transiently restore dystrophin in the myocardium and skeletal muscle of dystrophic mice, potentially via delivery of miRNA-148a [27].

MuSC-EVs may provide therapeutic benefits for myopathies and may help overcome many of the limitations of traditional MuSC-mediated therapeutic approaches; however, limited information exists regarding MuSC-EVs. Therefore, the purpose of this investigation was to characterize MuSC-EVs and examine potential therapeutic benefits in vitro. Importantly, mitochondrial damage has been causatively linked to impaired MuSC function [28,29], and impaired mitochondrial function is known to play a role in muscle dysfunction in models of muscular dystrophy [30,31,32,33], cancer cachexia [34], disuse atrophy [9,35,36], mechanical ventilation-induced diaphragm weakness, [9,37,38,39,40,41,42], hyperthermia [33], sarcopenia, [43,44], diabetes [12,45], and chronic kidney disease [46,47]. With the established links between MuSC function and mitochondrial function [48], we also investigated the extent to which MuSC-EVs could attenuate mitochondrial dysfunction. We hypothesized that MuSCs release an abundance of EVs that rapidly enter myotubes and attenuate oxidative stress-induced mitochondrial dysfunction.

## 2. Materials and Methods

### 2.1. Animals

All animal procedures were approved by the Institutional Animal Care and Use Committee at the University of Delaware under animal use protocol 1334 on 15 October 2018. C57BL/6 mice (N = 8, 4–6 weeks of age, mixed male and female) were anesthetized using isoflurane gas throughout the dissection protocol.

### 2.2. Isolation of MuSCs

All the muscles of the lower limb were removed from both limbs and stored on ice in wash buffer, consisting of 5% horse serum and 1% penicillin-streptomycin in Dulbecco’s Modified Eagle Media (DMEM). The muscles were minced into a slurry, containing pieces approximately 3 mm in diameter [49]. The minced tissue was transferred to dissociation buffer (3.3 mg/mL collagenase II in wash buffer) and incubated at 37 °C for 1 h while being mixed every 5–10 min. This was followed by a wash step and centrifugation at 500× *g* for 5 min before removing the dissociation buffer and adding stock collagenase II (4.3 mg/mL) (Worthington, Lakewood, CA, USA) and stock dispase (6.0 mg/mL) (Gibco, Gaithersburg, MD, USA) to the solution. This solution was incubated for an additional 30 min at 37 °C while mixing. A 10 mL, 20-gauge needle was used to triturate the solution. This was followed by an additional wash before the solution was run through a 40 µm filter. Two washes through the filter were performed. The cells were pelleted by centrifugation at 500× *g* for 5 min at 22 °C and all of the media was aspirated off. MuSCs were isolated from cell suspension via magnetic separation using the Satellite Cell Isolation Kit from Miltenyi Biotec (Bergisch Gladbach, Germany). We chose this method as previous studies have verified this kit results in a highly purified MuSC population [50,51,52].

### 2.3. MuSC Culture

Following isolation, the MuSCs were seeded on Matrigel-coated (Corning, Corning, USA) 6-well plates at a seeding density of 1.5 × 10^4^–2.0 × 10^4^ cells/cm^2^. The cells were grown in vesicle-free expansion media, consisting of 20% vesicle-free fetal bovine serum, 1% penicillin-streptomycin, 5 ng/mL basic-fibroblast growth factor (Progen, Heidleberg, Germany), and equal parts DMEM and Ham’s F10 mix. The MuSCs were cultured for six days in a 37 °C incubator with 5% CO_2_, with media being replaced and collected each day.

### 2.4. C2C12 Cell Culture

C2C12 myoblasts were cultured in various vessels depending on downstream application using methods we have previously described [13,21,53]. The myoblasts were seeded at a density of 1.0 × 10^4^ cells/cm^2^ and were then grown in a 37 °C incubator with 5% CO_2_. Myoblast growth media contained 10% fetal bovine serum and 1% penicillin-streptomycin in Dulbeccoe’s modified eagle media. The myoblasts were proliferated until they were 80–90% confluent before being switched to differentiation media containing 2% horse serum and 1% penicillin-streptomycin in Dulbeccoe’s modified eagle media. Following 5 days in differentiation media, for 5 days to allow full differentiation into myotubes. Fully differentiated myotubes were used in all EV experiments.

### 2.5. Nanoparticle Tracking Analysis

A Nanosight NS300 (Malvern Panalytical, Malvern, UK), equipped with a 532-nm green laser and NS300 FCTP Gasket (Cat no. NTA4137), was utilized for characterizing size and number of circulating exosomes. All samples were analyzed at a camera level of 12 and a detection threshold of 3 Video capture settings were set to record three videos for one min each [54]. Data were analyzed using NTA software v3.2. SC-EV samples were prepared with a 1:140 dilution in a total volume of 1 ml of sterile, filtered phosphate-buffered saline (PBS) and injected using a 1 mL sterile BD Plastipak syringe (Becton Dickinson S.A., Madrid, Spain).

### 2.6. Transmission Electron Microscopy (TEM)

Approximately 4.70 × 10^9^ EVs from the first day of MuSC culture and 4.12 × 10^10^ EVs pooled from days 2–6 of culture were resuspended in 1× PBS and used for TEM imaging. 400 mesh carbon-coated copper grids were floated onto drops of the sample. The grids were washed on four drops of water and then negative stained with 2% uranyl acetate (aq). Just prior to application of the sample, grids were glow discharged using a Pelco easiGlow glow discharge unit to render the carbon film hydrophilic. Samples were imaged using a Zeiss Libra 120 transmission electron microscope operating at 120 kV. Images were acquired using a Gatan Ultrascan 1000 CCD camera.

### 2.7. MuSC-EV Isolation, Labeling, and Uptake

EVs were isolated from MuSC culture media using ExoQuick TC (System Bioscience, Palo Alto, USA), following the manufacturer’s instructions and resuspended in 1× phosphate-buffered saline (PBS) for long term storage at −80 °C. A total of 1.8 × 10^11^ isolated MuSC-EVs were labeled using 10 µM carboxyfluorescein succinimidyl ester (CFSE) dye in PBS for 2 h at 37 °C. Following the incubation period, ExoQuick TC was used via manufacturer’s instructions to re-pellet and isolate the labeled MuSC-EVs. The labeled MuSC-EVs were then resuspended in 100 μL PBS and 50 µg labeled MuSC-EVs were added to the media of C2C12 myotubes in an eight-chamber cover glass slide and incubated at 37 °C for 24 h. Following the 24 h incubation period, the myotubes were washed with PHEM buffer (36.28 g PIPES, 11.92 g HEPES, 7.6 g EGTA, 1.97 g MgSO_4_ · 7H_2_O) to remove any labeled protein that was not incorporated into the myotubes and fixed in 4% paraformaldehyde (PFA) for 10 min at room temperature. Imaging of the MuSC-EV protein uptake was performed on the Leica LSM-880 confocal microscope system using the 488 nm laser line and 63× objective lens (Zeiss, Oberkochen, Germany) to obtain a representative image of labeled MuSC-EV protein uptake in the myotubes. 

To quantify the uptake of MuSC-EVs, C2C12 myotubes were cultured in a 96-well micro-clear plate (Greiner Bio-One, Monroe, LA, USA). Separately, EVs were isolated from MuSC culture media and labeled via incubation with 10 µM CFSE at 37 °C for 2 h. Excess dye was removed by re-isolating the labeled-EVs using ExoQuick TC. 1.00 × 10^9^ labeled EVs were then incubated in each well of the 96-well plate for 5 min, 30 min, 1 h, 2 h, 6 h, 24 h, and 48 h. Preliminary data from our lab found 1.00 × 10^9^ fluorescently labeled EVs added per well of differentiated myotubes in a 96-well plate (i.e. 3.13 × 10^9^ EVs per cm^2^ well surface area) resulted in significant uptake of EVs by the myotubes as measured by fluorescent intensity in the myotubes (data not shown). Further, significant levels of fluorescence were detected at various times points and thus 3.13 × 10^9^ EVs per cm^2^ plate surface area was chosen for the in vitro uptake experiments. Following the incubation period, the myotubes were washed with PHEM buffer to remove any labeled protein that was not incorporated into the myotubes and fixed in 4% PFA for 10 min at room temperature. The average spot intensity measurement on the CellInsight CX7 (Thermo Fisher Scientific, Waltham, MA, USA) was used to analyze the uptake of CFSE fluorescently labeled MuSC-EV protein delivered to C2C12 myotubes. The 488 nm laser line and 20× objective lens on the Leica LSM-880 confocal microscope system were used to obtain representative images of CFSE-labeled MuSC-EV protein in the myotubes at each timepoint.

### 2.8. MuSC-EV Protein Colocalization with Mitochondria

CFSE-labeled MuSC-EVs were incubated in the differentiation media of C2C12 myotubes for 24 h, as described in the previous section. Following the 24 h incubation period, the media was changed to media containing MitoTracker (Invitrogen, Carlsband, CA, USA) at a final concentration of 300 nM for 45 min in a 5% CO_2_ cell culture incubator. The media was then removed and the myotubes were washed and fixed in 4% paraformaldehyde solution. The myotubes were imaged on the Leica LSM-880 confocal microscope system using the 488 nm and 561 nm laser lines and 40× objective lens to obtain a representative image of MuSC-EV protein and mitochondrial colocalization.

### 2.9. Live Cell Metabolic Assay

C2C12 myotubes were cultured on eight-well Seahorse culture plates (Agilent, Santa Clara, CA, USA) and plated at 1.0 × 10^4^ cells per well (~9.4 × 10^4^ cells/cm^2^) in GM for 24 h before being switched to DM for 4 days prior to treatments. The wells on each plate were divided into four different groups: control (no treatment), MuSC-EV treatment (3.12 × 10^8^ SC-EV/well), treatment with 50 µM H_2_O_2_, or H_2_O_2_ with subsequent MuSC-EV treatment (H_2_O_2_ + SC-EV). Each treatment was conducted for 24 h. The H_2_O_2_ treatments were administered first by diluting the H_2_O_2_ in DM and performing a media change from regular DM to the H_2_O_2_-treated DM. Meanwhile, the untreated control and MuSC-EV treatment groups remained in regular DM. Following 24 h of H_2_O_2_ treatment, all groups received a wash with fresh DM as well as a media change with either regular DM or MuSC-EV treatment DM. The MuSC-EV and H_2_O_2_ + MuSC-EV groups received MuSC-EV treatment DM containing 3.12 × 10^8^ MuSC-EVs/well, whereas the untreated control and H_2_O_2_ groups received fresh regular DM containing no MuSC-EVs. The number of EVs/well was normalized based on surface area using the quantity derived during the previous uptake experiments (as described above). All conditions were performed in 3 independent experiments in duplicate (3 technical replicants). Following the treatment period, the media was removed, the cells were washed with XFp Assay media and then replaced with fresh XFp Assay media composed of 7.4 pH XF DMEM (Agilent, Santa Clara, CA, USA), supplemented with 1 mM pyruvate, 2 mM glutamine, and 10 mM glucose. The cells were incubated in the assay media for 45 min in a CO_2_-free 37 °C incubator and analyzed on the Agilent Seahorse platform following the manufacturer’s mitochondrial stress test protocol. Briefly, the compounds in the kit were reconstituted to the manufacturer’s specified concentrations: 1.5 µM Oligomycin, 1.0 µM 2-[2-[4-(trifluoromethoxy)phenyl]hydrazinylidene]-propanedinitrile (FCCP), and 0.5 µM rotenone A. Pre-specified amounts of these compounds were then loaded into the ports of the extracellular flux cartridge provided in the kit. The machine then exposed the myotubes to each compound in a sequential order while measuring various parameters of mitochondrial function.

### 2.10. Proteomic Analysis

Global proteomic analysis was performed by Bioproximity LLC (Manassas, Virginia, USA). Briefly, approximately 2.35 × 10^10^ MuSC-EVs resuspended in 1× PBS were analyzed via ultraperformance liquid chromatography-tandem mass spectrometry (UPLC MS/MS) using ThermoEasy-nLC 1200 fitted with a heated Easy-Spray column and Thermo Q-Exactive HF-X quadrupole-Orbitrap mass spectrometer. Peptide identification was performed with Comet and Tandem, using spectral library searching and match-between-runs algorithms. Quantitation was performed via MS1 peak measurement. Enrichment analysis was performed using the database DAVID (v6.8) to analyze the data for significant enrichment (false detection rate (FDR) < 0.05) of Gene Ontology (GO) terms in the categories: Biological Processes, Molecular Function and Cellular Component as well as Kyoto Encyclopedia of Genes and Genomes (KEGG) pathway analysis [55,56]. A threshold was then applied to the data to remove all GO and KEGG terms with a false detection rate (FDR) > 0.05. The data was then organized by the number of proteins identified for each significantly enriched GO/KEGG term in ascending order of FDR value.

### 2.11. Statistical Analyses

When results from two groups were compared, a t-test was used to test for significance. When results from more than two groups were compared, a one-way ANOVA was used to determine overall significance. When appropriate, a post hoc Tukey honestly significant difference test was performed. Differences in results were considered significant when *p* ≤ 0.05. For each outcome, at least 3 samples per treatment group, acquired from ≥3 independent experiments, were quantified and analyzed.

## 3. Results

### 3.1. Characterization of MuSC-EVs

**Figure 1:**
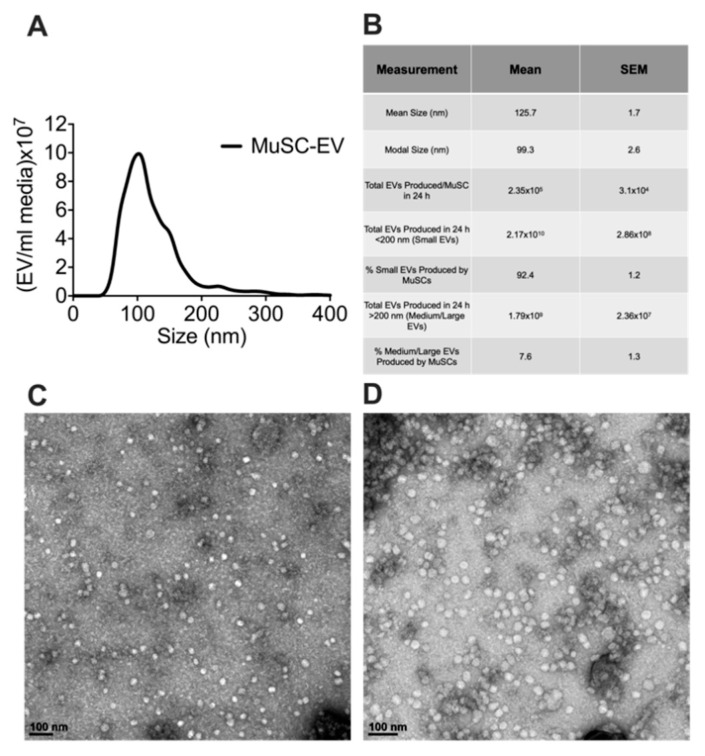
Characterization of MuSC-EVs. (**A**) Nanoparticle tracking analysis of extracellular vesicles (EVs) released during the first 24 h of SC culture, demonstrating the concentration and size profile of SC-EVs released in vitro. (**B**) Table of MuSC-EV characterization data. (**C**) Image of MuSC-EVs released during the first 24 h of cell culture taken via TEM. (**D**) Image of pooled MuSC-EVs released during days 2–6 of cell culture taken via TEM. N = 3 (biological replicates).

To determine the size and number of extracellular vesicles (EVs) released from MuSCs, media was collected from cultured MuSCs 24 h after plating cells (1.0–2.0 × 10^4^ cells/cm^2^). EVs were isolated from the media and characterized via nanoparticle tracking analysis (NTA). Over this 24 h period each MuSC released 2.35 × 10^5^ ± 3.10 × 10^4^ EVs, calculated using data derived from NTA and seeding density of the MuSCs. Since numerous EV subpopulations exist, we examined the size distribution of the MuSC-EVs. Our data show MuSC-EVs have a mean size of 125.7 ± 1.7 nm and modal size of 99.30 ± 2.6 nm, indicating MuSCs primarily release EVs in the size range of small and medium-sized EVs (Figure 1A,B). Further, to visualize the morphology of the EVs, TEM was performed on a subset of MuSC-EVs (Figure 1C,D).

### 3.2. MuSC-EVs Rapidly Deliver Protein into Myotubes

**Figure 2:**
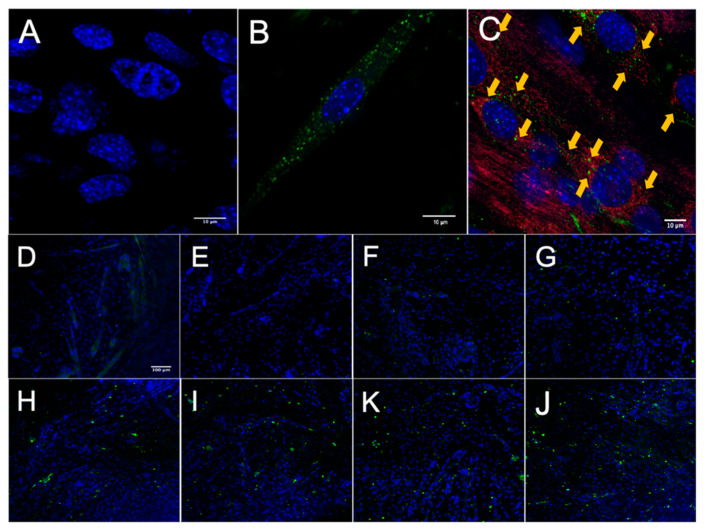
**Fluorescence Microscopy of MuSC-EV Protein Uptake in Myotubes.** (**A**) 40× negative control (No MuSC-EV treatment) of C2C12 myotubes with DAPI stain (blue). (**B**) Representative 63× image of carboxyfluorescein succinimidyl ester (CFSE)-labeled SC EV protein (green) delivery to C2C12 myotubes following 24 h incubation period in vitro. (**C**) Representative 40× image of CFSE-labeled MuSC-EV protein (green) delivery into C2C12 myotubes with mitochondrial stain (red) suggesting interaction of MuSC-EV protein with portions of the mitochondrial network. (**D**–**J**) 20× images of C2C12 myotubes incubated with 1 × 10^9^ CFSE-labeled SC-EVs/well in a 96-well plate for varying time periods. (**D**) Negative control (**E**) 5 min (**F**) 30 min (**G**) 1 h (**H**) 2 h (**I**) 6 h (**K**) 24 h (**J**) 48 h of incubation in vitro. N = 3 (technical replicates).

**Figure 3:**
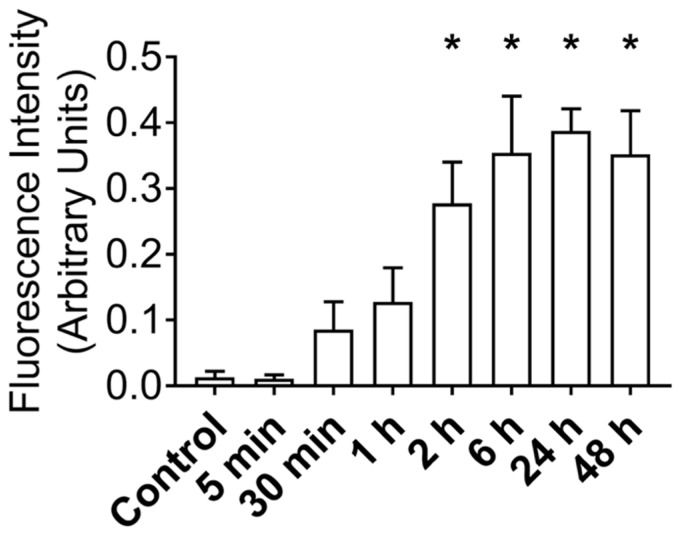
Fluorescence Intensity of CFSE-Labeled MuSC-EV Protein Delivered to C2C12 Myotubes In Vitro Following Varying Incubation Periods. * *p* < 0.05 vs. control. N = 3 (technical replicates).

After establishing that MuSCs release a substantial amount of EVs, we worked to demonstrate that these EVs could deliver proteins to recipient cells. Importantly, for these experiments we used differentiated C2C12 myotubes as recipient cells to better model adult muscle fibers, in vivo. MuSC-EVs were incubated with carboxyfluorescein succinimidyl ester (CFSE) to label the amino ends of all proteins present in the sample and were delivered to myotubes by adding the labeled EVs directly to the culture media. Following a 24 h incubation, myotubes were visually inspected using fluorescent microscopy. There appeared to be an abundance of fluorescent puncta within the cultured myotubes, suggesting the cells were able to uptake the CFSE-labeled protein from the MuSC-EVs (Figure 2B). Using the same technique previously described, we next quantified the kinetics of MuSC-EV protein delivery by incubating MuSC-EVs in the culture media of myotubes for various durations ranging from 5 min to 48 h. Fluorescence from the CFSE dye was then quantified to measure the amount of protein uptake in the myotubes. Remarkably, significant delivery of MuSC-EV protein was detected within the myotubes as early as 2 h after exposure (*p* = 0.0141; Figure 2H) and for upwards of 48 h after exposure (*p* = 0.0071; Figure 2K and Figure 3), indicating the cargo was not rapidly degraded once delivered into the myotubes. The greatest quantity of labeled protein was observed at 24 h after exposure (*p* = 0.0004; Figure 2J). All groups were compared to untreated control myotubes.

### 3.3. MuSC-EV Protein Colocalizes to Mitochondria in Myotubes

Once we established MuSC-EV protein uptake was greatest at 24 h, we next examined the localization of the protein inside the myotubes. CFSE-labeled MuSC-EVs were incubated in culture media of myotubes for 24 h. The myotubes were then stained with a mitochondria-specific dye. Fluorescent microscopy revealed what appeared to be overlapping signal (yellow) between a portion of the labeled MuSC-EV protein (green) and mitochondria (red) as well as fluorescent puncta juxtaposed to portions of the mitochondrial network (Figure 2C). The overlapping and juxtaposed signal suggested the MuSC-EV protein cargo may interact with portions of the mitochondrial network.

### 3.4. MuSC-EVs Reverse Peroxide-Induced Mitochondrial Dysfunction

**Figure 4:**
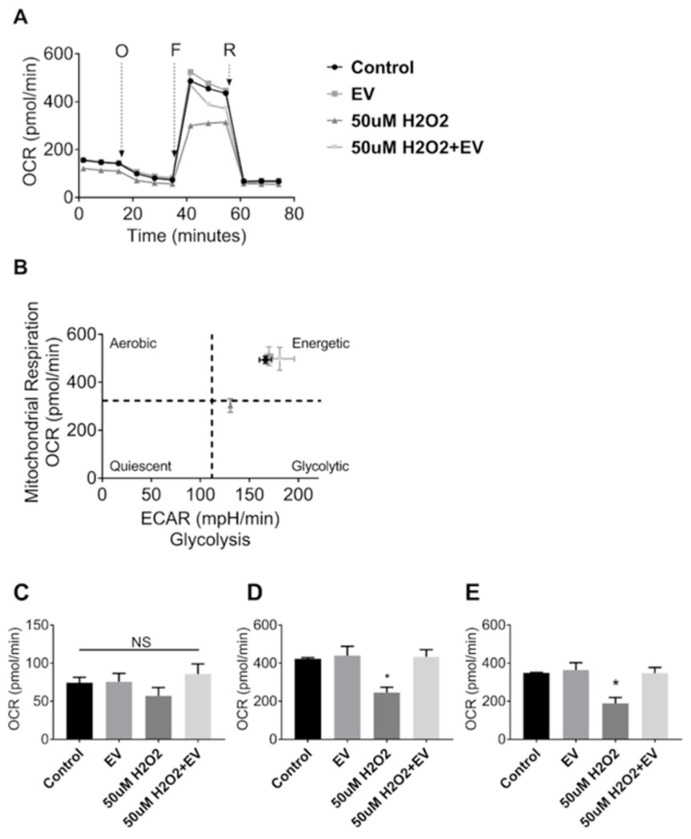
Mitochondrial Stress Test in C2C12 Myotubes. Myotubes were divided into one of four groups: untreated control, treatment with 3.12 × 10^8^ SC-EV for 24 h, treatment with H_2_O_2_ for 24 h, or treatment with H_2_O_2_ for 24 h followed by treatment with SC-EV for 24 h. (**A**) Oxygen consumption rate over the duration of the mitochondrial stress assay. O = oligomycin, F = 2-[2-[4-(trifluoromethoxy)phenyl]hydrazinylidene]-propanedinitrile (FCCP), R = Rotenone and Antimycin A. (**B**) Energy map depicting reliance on aerobic vs. glycolytic energy systems and energetic state of each treatment group. (**C**) Basal respiration of myotubes in each treatment group. (**D**) Maximal respiration of myotubes in each treatment group. (**E**) Spare respiratory capacity of myotubes in each treatment group (calculated as difference between maximal respiration and basal respiration). * *p* < 0.05. N = 3 (technical replicates).

Since MuSC-EVs effectively delivered protein to recipient muscle cells, we next explored the potential of these EVs to attenuate dysfunction caused by acute oxidative stress. Mitochondrial respiration was measured in C2C12 myotubes under control conditions and following a 24 h treatment with 50 µM hydrogen peroxide (H_2_O_2_). H_2_O_2_ exposure resulted in a 42% decline in maximal oxygen consumption rate (OCR) (*p* = 0.0243; Figure 4A,D) as well as a 46% reduction in spare respiratory capacity relative to the untreated control group (*p* = 0.0185; Figure 4A,E). Subsequent treatment with MuSC-EVs (3.12 × 10^8^ SC-EV; 24 h) following H_2_O_2_ exposure resulted in a 76% increase in maximal OCR (*p* = 0.0187; Figure 4A,D) and 84% increase in spare respiratory capacity in the damaged myotubes (*p* = 0.0198; Figure 4A,E), reversing H_2_O_2_-induced dysfunction. We also included myotubes treated with MuSC-EVs without oxidative insult. Maximal OCR and spare respiratory capacity were similar to control myotubes (Figure 4D,E). Further, the ratio of oxygen consumption rate to extracellular acidification rate indicates that treatment with MuSC-EVs restored the energetic phenotype of the damaged myotubes to that of the control group (Figure 4B). There were no significant differences between the basal respiration values of each group (Figure 4C).

### 3.5. Proteomic Analysis of MuSC-EVs

**Figure 5:**
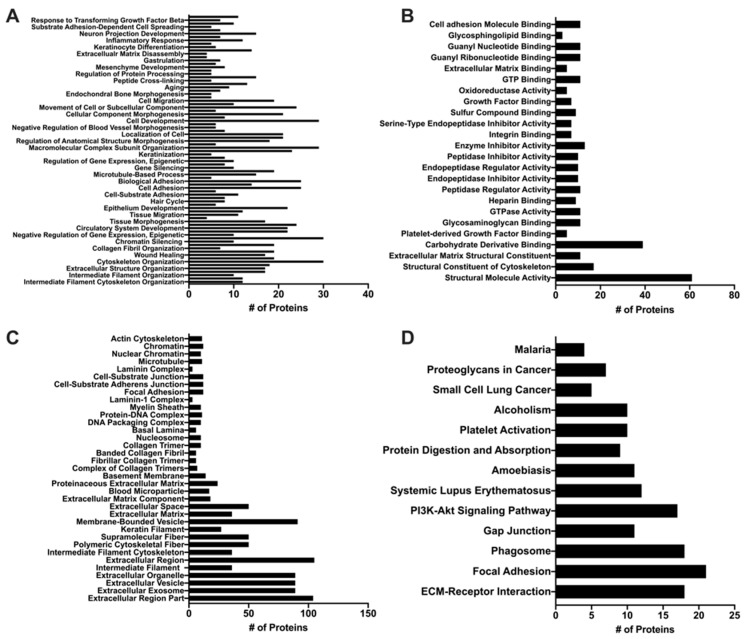
Proteomic Analysis of MuSC-EVs. Significantly enriched (*p* < 0.05) Gene Ontology (GO) and Kyoto Encyclopedia of Genes and Genomes (KEGG) terms identified using DAVID enrichment analysis following UPLC MS/MS of MuSC-EVs. (**A**) Biological processes (**B**) Molecular function (**C**) Cellular component (**D**) KEGG Pathway results.

Given the ability of MuSC-EVs to reverse oxidatively-induced mitochondrial dysfunction in myotubes, we next sought to determine the contents of the EVs in attempt to gain insight as to how they mediate these effects. UPLC MS/MS was utilized to identify the protein signature of a sample of MuSC-EVs produced during the first 24 h of MuSC culture. A total of 423 proteins were identified in the sample, 129 of which were associated with DAVID IDs. Enrichment analysis revealed the significantly enriched (FDR < 0.05) GO and KEGG terms associated with the proteins identified by UPLC MS/MS. 309 GO terms were identified for biological processes, 80 of which were significantly enriched, with intermediate filament organization presenting the lowest FDR value (4.59 × 10^−12^) and cytoskeleton organization and single-organism organelle organization presenting the highest count values (30 identified proteins) (Figure 5A). Overall, 62 GO terms were identified under molecular function, 25 of which were significantly enriched, with structural molecule activity presenting the lowest FDR value (1.5 × 10^−45^) and carbohydrate derivative binding presenting the highest count value (39 identified proteins) (Figure 5B). Overall, 60 GO terms were identified under cell component, 35 of which were significantly enriched, with extracellular region part presenting the lowest FDR value (1.29 × 10^−37^) and extracellular region presenting the highest count value (105 identified proteins) (Figure 5C). Additionally, 14 KEGG pathways were identified, 13 of which were significantly enriched, with extracellular matrix (ECM)-receptor interaction presenting the lowest FDR value (1.08 × 10^−17^) and focal adhesion presenting the highest count value (21 identified proteins) (Figure 5D).

## 4. Discussion

Use of MuSCs as therapeutic agents has been widely investigated [2,7,57]. The therapeutic application of MuSCs is not completely understood but is largely attributed to their ability to differentiate into myoblasts and fuse into damaged fibers to support regeneration [6]. However, MuSC-mediated therapy is hindered by several major limitations, including the number of MuSCs needed to treat systemic muscle diseases and the loss of myogenic potential during ex vivo expansion [2,14,15,16]. Interestingly, mesenchymal stem cells (MSCs) release EVs that confer many of the same regenerative benefits as the cells themselves [58]. Further, typically cells continually release EVs and thus from a therapeutic standpoint EVs may provide a more efficient and scalable production method than cell-based therapeutics. Additionally, EVs have been shown to efficiently deliver functional molecular cargo into cells [59,60,61]. Therefore, MuSC-EVs could feasibly overcome the major limitations of MuSC-based therapeutic approaches. The purpose of this investigation was to characterize MuSC-EVs and evaluate the extent to which MuSC-EVs can aid in reversing hydrogen peroxide-induced muscle damage. 

Calculations in the present study show approximately 2.35 × 10^5^ EVs were released per MuSC per 24 h of culture. To our knowledge, this is the first study quantifying the number of EVs released per MuSC per 24 h. However, most studies quantifying number of EVs present the data as EVs per milliliter of media or bodily fluid. This is likely due to nanoparticle tracking analysis platforms quantifying data as particle per milliliter. Reporting the data this way makes it difficult to compare the quantity of EVs released from a specific cell line under differing conditions. Back calculations can be performed on studies that report cell number, amount of time, and concentration of EVs. 

However, even when back calculating for number of EVs released per cell per 24 h, comparing number of EVs released both by other stem cell types and other cell lines presents several challenges regarding EV isolation and cell culture methods [62]. For example, studies reporting the number of EVs released from MSCs vary widely when back calculating to number of EVs released per cell per 24 h of culture [63,64]. This is also the case when examining the number of EVs released from other cell types, such as human umbilical vein endothelial cells and human embryonic kidney cells [65,66]. Therefore, it is evident the use of varying isolation and cell culture methodologies as well as varying cell types result in different numbers of EVs obtained during cell culture. To accurately make a comparison of EVs released based on cell type, one must control for isolation and culture methods and implement a standardized approach of reporting EV release data. In addition to variations in the number of EVs obtained, the isolation method utilized may also impact the identity and cargo of the EVs. In the current study, MuSC-EVs were isolated using a precipitation based method (ExoQuick TC from System Bioscience, Palo Alto, CA, USA). Compared to ultracentrifugation, precipitation methods and kits using precipitation methods have been shown to result in both increased number of EVs as well as higher extraction efficiency and purity of small RNA in EVs isolated from cell culture media [67]. Precipitation-based methods have also been shown to yield an overall greater number of EVs and higher protein concentration than ultracentrifugation [68].

Our data suggest MuSCs produce an abundance of EVs that deliver large amounts of cargo into myotubes, some of which colocalizes to intracellular mitochondria. To follow up this finding, we investigated the effects of MuSC-EVs on mitochondrial function. Our data demonstrate MuSC-EVs reverse mitochondrial dysfunction caused by oxidative stress. Mitochondria are critical in cellular energy production as well as the regulation of overall cellular viability [69]. Mitochondrial dysfunction plays a central role in numerous muscle disorders and pathologies, including Duchenne Muscular Dystrophy (DMD) [30,70]. For example, work by Hughes et al. (2019) demonstrated that mitochondrial dysfunction, attributed to impaired oxidative phosphorylation and elevated oxidant production, contributes to early stage pathology in the D2.mdx mouse model [21]. Additionally, in the mdx mouse mode mitochondrial dysfunction is one of the earliest cellular consequences of DMD and is associated with the impaired ability of dystrophic muscle cells to respond to sarcolemmal damage [70]. Adverse alterations in mitochondrial morphology and function are also known to play a central role in the pathology of muscle dysfunction and wasting in murine models of cancer cachexia, attributed to decreased adenosine triphosphate production and uncoupling of oxidative phosphorylation [71]. The contribution of mitochondrial dysfunction to disuse atrophy is well documented [35]. For example, work by Powers et al. (2011) demonstrated a mitochondrial-targeted antioxidant was able to ameliorate ventilation-induced diaphragm weakness [37]. Given that mitochondrial dysfunction is associated with or contributes to an array of muscle pathologies, MuSC-EVs may alleviate a significant disease burden and provide therapeutic relief to a large number of patients. MuSC-EVs may have distinct advantages over cell transplantation including, most notably, the abundance of EVs released and the efficiency with which they deliver molecular cargo.

While it is clear that MuSC-EVs attenuate mitochondrial dysfunction caused by acute oxidative stress, the underlying mechanism is decidedly less apparent. Speculatively, these EVs may deliver some factor (RNA and/or protein) that serves to reverse H_2_O_2_-mediated mitochondrial damage and restore mitochondrial function. However, the identity of this factor or factors remains unknown. Given the 24 h period of MuSC-EV exposure to myotubes there is ostensibly sufficient duration to allow translation of delivered RNA or incorporation of delivered protein into the appropriate cellular compartments, including the mitochondria themselves.

To provide context to our data demonstrating the ability of MuSC-EVs to efficiently deliver fluorescently-labeled protein cargo and reverse mitochondrial dysfunction in myotubes in vitro, we analyzed a sample of MuSC-EVs via UPLC MS/MS. As previously mentioned, the identified list of proteins was analyzed via enrichment analysis using the database DAVID for significant enrichment of GO terms falling under biological processes, molecular function and cell component, as well as KEGG pathways. The top GO terms (lowest FDR value) under biological processes represent processes involving the regulation and organization of structural proteins, such as intermediate filament organization, extracellular structure organization, and cytoskeleton organization. These findings are particularly interesting in light of previous work by Fry et al. (2017) [22] and Murach et al. (2020) [25] demonstrating miRNAs, such as miR-206, contained within MuSC-EVs regulate extracellular matrix (ECM) remodeling. The top molecular function GO terms also reflect proteins involved in the regulation and organization of the cytoskeleton and ECM, with structural molecule activity, structural constituent of the cytoskeleton and ECM, and structural constituent as the top results. Given our data demonstrating the involvement of MuSC-EVs in regulating metabolic processes in myotubes, two other interesting molecular function GO terms that were significantly enriched are carbohydrate derivative binding and oxidoreductase activity. In accordance with the functions associated with identified proteins, the top cell component result was the GO term extracellular region part. The cell component results also correctly identified the proteins as being associated with EVs. The top KEGG pathways were ECM-receptor-interaction and focal adhesion further suggesting a role of the MuSC-EV proteins in ECM regulation and organization. Other interesting pathways that appear in the results are the phosphoinositol-3-kinase/Akt (PI3K/Akt) and phagosome pathways. These may provide interesting mechanistic insights, given that these pathways regulate critical anabolic (PI3K/Akt) [72] and catabolic (phagosome) [73] processes in myotubes. In addition to the enrichment analysis data, which analyzes the identified proteins more broadly based on function and localization, we identified a particularly interesting protein in the dataset in relation to our discoveries regarding MuSC-EVs and mitochondrial function. We identified peroxisome proliferator-activated receptor gamma coactivator 1-beta (PGC1-beta), which activates the transcriptional activity of transcription factors, such as estrogen-related receptor and nuclear respiratory factor, promoting mitochondrial biogenesis [74]. Further, PGC1-beta has been shown to coordinate with peroxisome proliferator-activated receptor gamma coactivator 1-alpha (PGC1-alpha) in the promotion of mitochondrial biogenesis [75]. This is a promising association as PGC1-alpha gene transfer has consistently attenuated disease severity and promoted a type I, oxidative phenotype in the skeletal muscle of mdx mice [76]. Based on the available data, we cannot be certain that this protein cargo indicates a definitive mechanism. However, these data provide important insights into the molecular cargo contained within MuSC-EVs when interpreted alongside the therapeutic effects of MuSC-EVs on mitochondrial function. Overall, these proteomics findings should be interpreted as a descriptive identification of potential targets for further, more definitive mechanistic investigations of the contents and therapeutic potential of MuSC-EVs.

Other groups have also shed light on potential mechanisms by which MuSCs and related stem cell types confer therapeutic effects beyond the well-documented fusion-mediated role of these cells. Fang et al. (2020) showed MuSC-derived IGF-2 induces an anti-inflammatory phenotype in macrophages, potentially due to modulation of oxidative phosphorylation [26]. This is a particularly interesting finding in line with our data demonstrating metabolic reprogramming in cultured myotubes. Gartz et al. (2019) demonstrated that exosomes from wild type and dystrophin-deficient induced pluripotent stem cell-derived cardiomyocytes exert cardioprotective effects on dystrophic cardiomyocytes [77]. The authors attributed these findings to a decrease in reactive oxygen species and delay in the formation of the mitochondrial transition pore following oxidative injury with H_2_O_2_, mediated by ERK1/2 and p38 MAPK signaling. Alternatively, MuSC-EVs may deliver intact mitochondria to recipient cells. This mechanism is attractive as EV-mediated delivery of mitochondria to damaged neurons has been demonstrated previously [78]. Our data indicate that MuSC-EVs delivered to undamaged myotubes did not alter mitochondrial respiration. If mitochondria were being delivered, we would expect to see increased respiration in this group. Given that there was only an increase in mitochondrial respiration in the group that was damaged first, it appears that the MuSC-EVs are delivering cargo that helps restore mitochondrial function but does not expand the overall mitochondrial capacity of otherwise healthy myotubes. In addition, there are a number of other cellular toxins, such as paraquat and rotenone [79], that are known to cause mitochondrial dysfunction and should be investigated to fully elucidate the therapeutic effects of MuSC-EVs as well as gain insight into potential mechanisms of action.

## 5. Conclusions

In summary, these data suggest that MuSCs release large amounts of EVs of multiple sizes which are able to deliver molecular cargo to mature myotubes. We also demonstrate that these MuSC-EVs reverse oxidative stress-mediated mitochondrial dysfunction and restore the energetic phenotype of the damaged myotubes. Given this profound therapeutic response and the role of oxidative stress in a host of muscle pathologies, MuSC-EVs may be an effective, simple, safe, and scalable therapeutic approach. Future studies are needed to investigate the physiological roles of MuSC-EVs in the treatment of these pathologies as well as an identification of the mechanisms that underlie MuSC-EV-mediated restoration of mitochondrial function.

## 6. Patents

Extracellular vesicles from satellite cells for treatment of myopathies. Inventors: M.B.H., J.T.S., B.E.W., K.T.S., E.R.M., A.D.M.
